# First implementation of photon-counting dector computed tomography for optimizing segmentation in head-and-neck cancer radiotherapy

**DOI:** 10.1016/j.phro.2025.100864

**Published:** 2025-11-13

**Authors:** Niccolò Bertini, Hubert S. Gabryś, Hatem Alkadhi, Lotte Wilke, Patrick Wohlfahrt, Serena Psoroulas, Eugenia Vlaskou, Laura Motisi, Matthias Guckenberger, Stephanie Tanadini-Lang, Panagiotis Balermpas

**Affiliations:** a Department of Radiation Oncology, University Hospital Zurich, University of Zurich, Rämistrasse 100, 8091 Zurich, Switzerland; b Radiation Oncology Unit, Oncology Department, Azienda Ospedaliero Universitaria Careggi, Florence, Italy; c Diagnostic and Interventional Radiology, University Hospital Zurich, University of Zurich, Zurich, Switzerland; d Siemens Healthineers, Varian, Cancer Therapy Imaging, Forchheim, Germany

**Keywords:** Head and neck cancer, Radiotherapy planning, Segmentation, computed tomography (CT), Photon-counting

## Abstract

**Background and purpose:**

Photon-counting detector computed tomography (PCD-CT) offers advancements over conventional energy-integrating detector computed tomography (EID-CT). This study is the first to investigate the use of PCD-CT for delineation purposes.

**Materials and methods:**

Twelve patients with head-and-neck cancer were prospectively included. Delineation of clinical target volume (CTV) and organs at risk (OARs) was performed by three physicians using EID-CT (120 kVp) and PCD-CT at virtual monoenergetic levels of 140 keV and 50 keV. Segmentation accuracy was assessed and contouring-confidence was rated.

**Results:**

The ratio of the mean computed tomography dose index (CTDI) for EID-CT/PCD-CT for all patients was 2.72. The study revealed high inter-observer agreement for the mandible, oral cavity, parotid glands, and submandibular glands, with DSCs exceeding 0.8 across both EID-CT and PCD-CT. No differences in delineation agreement were observed, while a significant improvement was found for the lips (p = 0.001) and oral cavity (p = 0.003) when combining 140  keV and 50  keV images in PCD-CT compared to EID-CT. PCD-CT outperformed EID-CT in terms of contouring confidence.

**Conclusion:**

The study established the feasibility of PCD-CT for radiotherapy planning. PCD-CT-based delineation provides results comparable to EID-CT, with the added advantages of reduced radiation dose, improved image quality and higher delineation confidence. Significant improvements in delineating specific structures, such as the lips and oral cavity, were observed when combining multiple energy levels in PCD-CT.

## Highlights

• PCD-CT provides enhanced image quality, reduced noise and artifacts, and lower radiation dose compared with conventional CT.
• This study is the first to investigate the use of PCD-CT for delineation purposes.
• Accuracy of organ and target segmentation between PCD-CT and energy-integrating detector computed tomography (EID-CT) was compared.
• The study established the feasibility of PCD-CT for radiotherapy segmentation.
• PCD-CT-based delineation is at least comparable to conventional EID-CT.

## Graphical abstract

**Figure:**
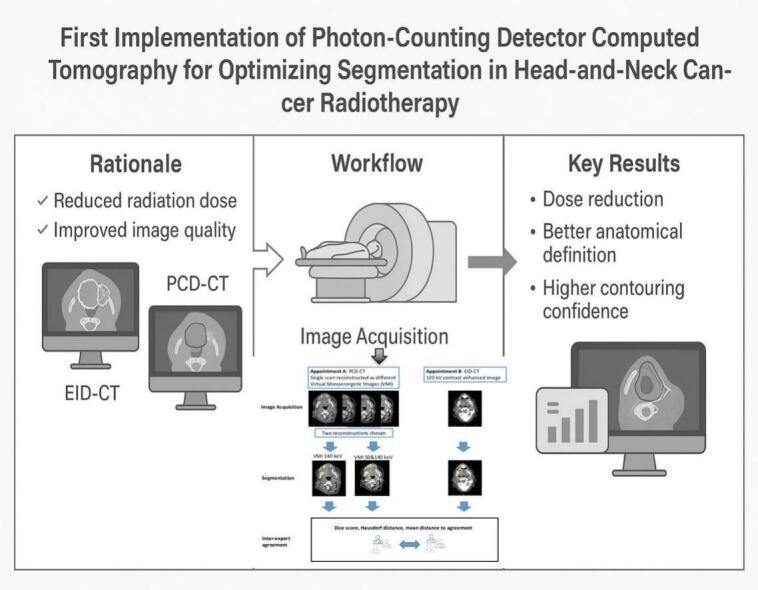

## 1 Introduction

Photon-counting detector computed tomography (PCD-CT) represents a substantial advancement in computed tomography technology [1,2]. PCD-CT offers several advantages over energy-integrating detector computed tomography (EID-CT), including reduced electronic noise, enhanced contrast-to-noise ratio, greater radiation dose efficiency, superior spatial resolution, and the ability to perform simultaneous multi-energy acquisition at a single x-ray tube potential [[3], [4], [5], [6], [7], [8], [9], [10], [11], [12], [13]]. These features make PCD-CT a promising technology, offering superior imaging quality and enhanced diagnostic information [11,12]. Additionally, PCD-CT may require lower doses of contrast media compared to EID-CT [[3], [4], [5], [6], [7], [8], [9], [10], [11], [12], [13]]. The simultaneous multi-energy data acquisition allows for advanced processing techniques, such as the creation of virtual non-contrast (VNC) reconstructions without additional exposure [[13], [14], [15], [16], [17], [18]].

For radiotherapy planning the advantages of PCD-CT could be of utmost relevance. EID-CT scans, commonly used in radiotherapy planning, are limited by electronic noise and lower spatial resolution, which can impact the precision of tumor and organs at risk (OARs) delineation. PCD-CT addresses these limitations, potentially leading to more precise delineation. Moreover, studies have highlighted PCD-CT's ability to improve the characterization of artificial materials, such as dental fillings, implants, and osteosynthesis materials, common in patients undergoing radiotherapy.

Despite these promising features and pioneering studies on using PCD-CT for radiotherapy purposes [[19], [20], [21], [22]] there is currently only scarce evidence specifically addressing the use of clinically acquired PCD-CT for organ and target segmentation in radiation oncology, even less for the challenging scenario of head and neck cancer. The studies mentioned above could demonstrate improved organ segmentation in abdominal phantoms [19] and at least equal accuracy of the quantitative information to this obtained by EID-CT [20]. Several studies also reported on the feasibility of implementing Dual Energy CT (DECT) for radiotherapy segmentation and planning, a technique also capable of reconstructing virtual monoenergetic images (VMI) [23,24].

Primary goal of this study is to demonstrate the feasibility of implementing PCD-CT for delineation purposes in challenging, real-life head and neck cancer cases, and to assess whether it can achieve at least similar agreement among clinicians when contouring OARs and targets compared to EID-CT.

## 2 Materials and methods

### 2.1 Patient cohort

The patient cohort consisted of 12 patients with head-and-neck cancer who had target volumes near radio-opaque oral implants. The latest included dental-based implants, extensive metal fillings (including amalgam) or titan plate reconstructions of the jaw after tumor surgery being within the CTV or within 2 cm of the CTV (Supplementary Fig. S6). Inclusion criteria were: patients referred for routine radiotherapy, completion of the PCD-CT examination, age over 18 years, and consent to the use of personal health data via the signed hospital‘s General Consent form. Exclusion criteria included pregnant or breastfeeding patients, those without radio-opaque artificial oral implants, and patients with incomplete CT data.

### 2.2 Patient characteristics

In this study, nine patients were male, three were female, with an average age of 63.9 years. Eleven tumors were squamous cell carcinomas (SSC); one was undifferentiated. The primary tumor location was predominantly in the oral cavity, with one case having a submental nodal metastasis. The staging distribution among the patients varied: one was classified as Stage III, 11 as IVa-IVc. Regarding the intent of radiotherapy, nine patients received adjuvant, two definitive, and one received palliative radiotherapy. A summary of patient and tumor characteristics is provided in Table 1.

**Table 1:** Patient and tumor characteristics.

Patients characteristics	Number
**Sex**	
- Male	9 (75 %)
- Female	3 (25 %)
**Age (years)**	
**mean**	63.9
**range**	45–90
**Tumor Histology**	
- scc	11 (91.67 %)
- Undifferentiated	1 (8.33 %)
**Primary tumor location**	
- Oral cavity	11 (91.67 %)
- Unknown (cervical CUP)	1 (8.33 %)
**Stage**	
- III	1 (8.33 %)
- IVa	7 (58.34 %)
- IVb	3 (25 %)
- IVc	1 (8.33 %)
**Intent of Radiotherapy**	
- Definitive	2 (16.67 %)
- Adjuvant	9 (75 %)
- Palliative	1 (8.33 %)
**Systemic treatment**	
- Yes	6 (50 %)
- No	6 (50 %)
**Type of systemic treatment**	
- Cisplatin	5 (42 %)
- Pembrolizumab	1 (8 %)
**Total**	12 (100 %)

All data was collected prospectively following approval by the institutional review board (number: 2022–00676). Only data collected during the standard clinical diagnostic process was included, ensuring no additional burden on the patients, as the EID-CT is needed for planning anyway and a timely diagnostic imaging (in this case PCD-CT) is always used in our department. No further study-specific procedures were performed.

**Fig. 1:**
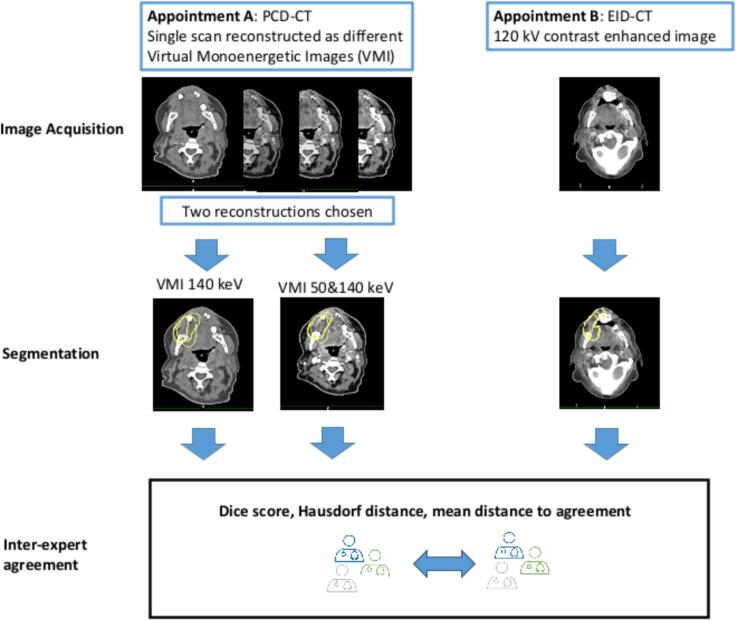
Workflow of the analysis: PCD-CT and EID-CT were acquired within one week. Two PCD-CT reconstructions (50 and 140 keV) were selected. Three certified radiation oncologists performed delineations on the EID-CT and both PCD-CT reconstructions. Pairwise Dice scores, Hausdorff distance, and mean distance to agreement were calculated for the CTV and OARs to assess and compare the inter-expert agreement across PCD-CT 50 keV, PCD-CT 140 keV, and EID-CT.

The workflow used in the image acquisition and data analysis is shown in Fig. 1.

Appointment A” was the day of the Photon-counting CT and “Appointment B” the one of the “conventional” planning CT. The two appointments had to be performed within one week from each other. All experts contoured all three images (PCD-CT 140 and 50 keV and EID-CT) for every patient on separate days. They were required to complete segmentation of one reconstruction type for all patients before proceeding to the next image type.

### 2.3 Imaging

All PCD-CT scans were performed at a Siemens NAEOTOM Alpha scanner (Siemens Healthineers) approved for clinical use in radiology. The patients were scanned using our institute standard radiology protocols, with 120 kVp, 500 ms exposure time, 200 mA tube current and on average 100 mAs exposure.

In PCD-CT, different reconstruction options are available, exploiting the multi-energy spectral information detected by the system. For this study, we selected two energy levels: 50 and 140 keV virtual mono-energetic images (VMI). Low-energy VMI (50 keV) offers good soft tissue and iodine contrast but can include much stronger artifacts for highly attenuating materials. On the other hand, high-energy VMI (110–140 keV) shows reduced soft tissue and iodine contrast, but in general less artifacts, also dependent on the specific material [[25], [26], [27]].

Different reconstruction algorithms among those available from Siemens Healthineers were evaluated. The Br40 kernel was selected for this study. The acronym is for 'Body regular', identifying a reconstruction kernel with slight edge enhancement, which is appropriate for delineation purposes. The '40' refers to the resolution index of the reconstruction, representing an intermediate resolution (“soft” image impression) that produced images optimal for soft tissue structures, as opposed to sharper kernels used for bones. As reference, we used the standard protocol for planning EID-CT (performed at a Siemens SOMATOM Definition AS) at our institute: a 120 kVp image (exposure 375 mAs, current 450 mA), performed on a Siemens Healthineers SOMATOM Definition AS scanner.

**Fig. 2:**
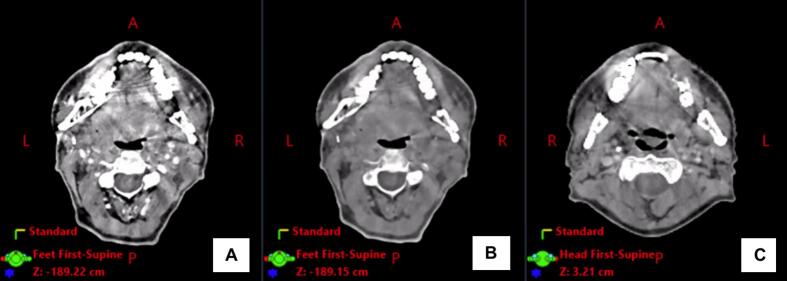
Head and neck images acquired with different CT techniques. From left to right: EID-CT, PCD-CT 50 keV, PCD-CT 140 keV.

The resolution of the EID-CT, PCD-CT 140 keV, and PCD-CT 50 keV was 1.27–1.52 mm, 0.39–0.66, and 0.47–0.60, respectively. The slice thickness for EID-CT was always 2 mm, whereas for PCD-CT it ranged from 1.5 to 3 mm. Example images are shown in Fig. 2.

### 2.4 Target and OARs delineation

As part of routine practice, target volumes, including Clinical Target Volume (CTV) and OARs, were delineated according to the guidelines [28], [29] on both the EID-CT and the PCD-CT images. Three experienced physicians performed these delineations using Varian Eclipse software.

The contoured OARs included the lips, mandible, oral cavity, pharyngeal constrictors, buccal mucosa, parotid glands and submandibular glands. Manual contours at 140 keV were created using only this energy level, while those at 50 keV were done by reviewing both 140 keV and 50 keV.

### 2.5 Contouring confidence rating

After performing contouring on both EID-CT and PCD-CT scans, all specialists evaluated the subjective contouring confidence of the following structures: 1) CTV, 2) mandible, 3) lips, and 4) other organs at risk, using a confidence scale ranging from 0 to 10. The mean values and range for each rating are reported.

### 2.6 Statistical analysis

For each region of interest (ROI), three segmentation metrics were calculated: Dice similarity coefficient (DSC), Hausdorff distance (HD), and mean distance to agreement (MDA). Because each patient was contoured using three different imaging modalities (EID-CT, PCD-CT at 140 keV, and PCD-CT 50 keV) and each case was contoured by three expert pairs, we used mixed-effects models with imaging modality as a fixed effect and patient and expert pair as random effects.

Since DSC values range from 0 to 1, we used a beta generalized linear mixed model with a logit link. Models were fit with the glmmTMB in R. Because HD and MDA are strictly positive and right-skewed, we used linear mixed-effects model with log-transformed outcomes, fit with the lmer package in R.

For each ROI and modality, estimated marginal means (EMM) were calculated. To control the family-wise error (FWER) within each ROI, *p*-values were adjusted using the Holm method.

## 3 Results

### 3.1 Dose exposure

We calculated the mean computed tomography dose index (CTDI) for EID-CT and PCD-CT across all patients. The ratio of mean CTDI (EID-CT/PCD-CT) was 2.72, indicating an overall lower radiation dose with PCD-CT for the majority of patients (see supplementary figures S5a-S5c).

### 3.2 Agreement among experts

**Fig. 3:**
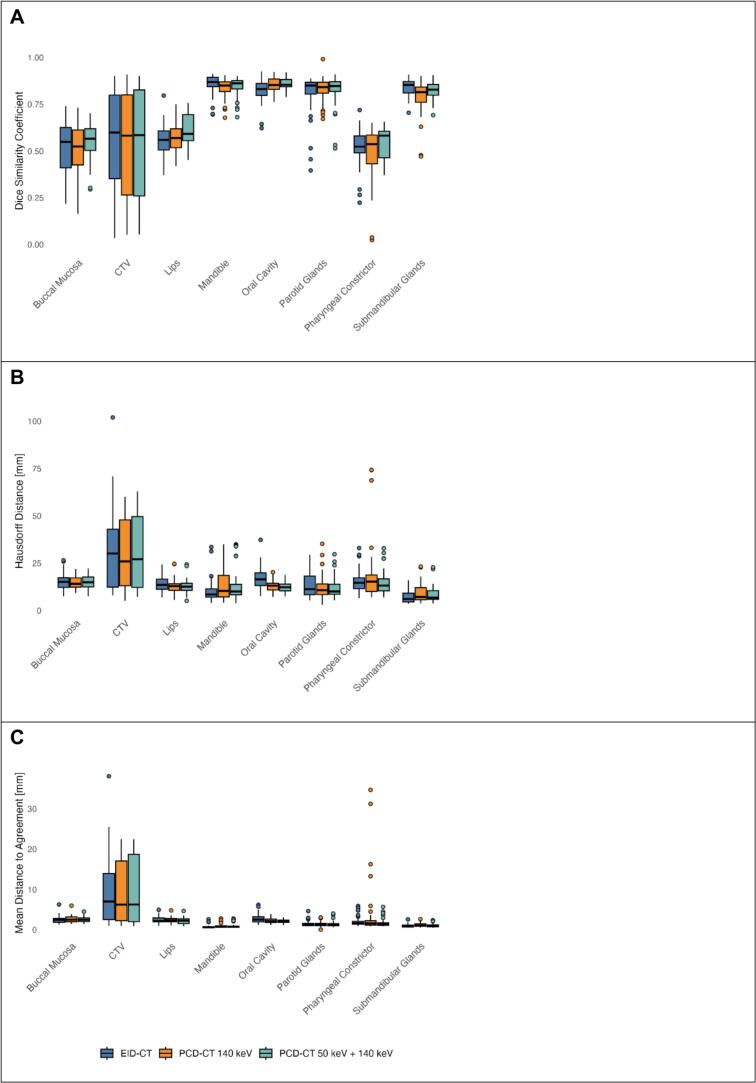
Boxplots showing inter-expert delineation agreement across imaging modalities: EID-CT, PCD-CT 140 keV, and combined PCD-CT 50 keV + 140 keV. Agreement was quantified using (A) Dice Score, (B) Hausdorff Distance, and (C) mean distance to agreement.

Inter-expert agreement in terms of DSC was high for the mandible, oral cavity, parotid glands, and submandibular glands, with values exceeding 0.8 across all imaging modalities (Fig. 3; Supplementary Table S1). In contrast, the CTV, buccal mucosa, lips, and pharyngeal constrictor showed lower agreement, with DSC values averaging around 0.5. MDA and HD were highest for the CTV (approximately 6 mm and 25 mm, respectively; Fig. 3B-C; Supplementary Table S2-3). For most other ROIs, HD values were similar, ranging from 10 mm to 15 mm, while the submandibular glands had lower HD values ca. 6 mm). MDA was smallest for the submandibular glands (ca. 1 mm) and the mandible (0.7 – 0.8 mm).

Among all ROIs, the CTV showed the greatest variability in DSC, HD, and MDA. No significant differences in segmentation agreement were observed between the imaging modalities overall. The most significant modality-specific differences were found for the lips, mandible, and oral cavity. For example, segmentation agreement for the lips was higher in PCD-CT at 50 keV than in EID-CT in terms of DSC (p = 0.001) and MDA (p = 0.047). Similarly, oral cavity segmentation showed higher agreement in PCD-CT at 50 keV and PCD-CT at 140 keV compared with EID-CT in terms of DSC (p = 0.003 and p = 0.016), HD (p < 0.001), and MDA (p = 0.005 and p = 0.022). Complete information on segmentation metric values and *p*-values is available in Supplementary Tables S1-3.

### 3.3 Contouring confidence rating

The mean confidence ratings across all observers showed a trend for higher confidence for all contoured volumes when using PCD-CT, especially for the lips and the CTV. Notably, the subjective rating was higher for all segmented volumes. Specifically, the mean scores (range) for EID-CT were: 1) CTV 7.3 (range 5–9), 2) mandible 9.2 (range 6–10), 3) lips 6.9 (range 4–9), and 4) other organs at risk 8.1 (6–10). For PCD-CT, the mean scores (range) were: 1) CTV 8.2 (range 6–10), 2) mandible 9.7 (range 8–10), 3) lips 8.1 (range 5–10), and 4) other organs at risk 8.8 (6–10). Detailed results are provided in Supplementary Table S4.

## 4 Discussion

In this study, we prospectively evaluated the feasibility of using photon-counting CT (PCD-CT) for delineation in radiation oncology, focusing on the challenging clinical scenario of head-and-neck cancer, where several OARs and the CTV are located near oral implants that cause imaging artifacts.

PCD-CT offers potential advantages in reducing radiation dose and improving ROI delineation in head and neck cancer. Our study showed that the ratio of the mean CTDI between EID-CT and PCD-CT was 2.72, confirming the overall lower dose with PCD-CT for most patients. Similarly, Jungblut et al. [30] reported that PCD-CT reduced the radiation dose by 66 % compared to energy-integrating detector CT (EID-CT), while maintaining diagnostic image quality in interstitial lung disease, with superior image sharpness and reduced noise.

Our findings indicate that most ROIs did not show a statistically significant differences in inter-expert delineation agreement between EID-CT and PCD-CT 140 keV. However, combining 140  keV and 50  keV reconstructions in PCD-CT led to significant improvements in the delineation of certain OARs, such as the oral cavity and lips. Interestingly, some volumes, such as the buccal mucosa and lips, showed lower agreement in terms of DSC and MDA with PCD-CT at 50 keV compared to EID-CT, while Hausdorff Distance (HD) values remained similar across modalities. This result is not surprising, considering the proximity of these structures to metal artifacts and the superior artifact reduction achieved with PCD-CT at 140 keV [31]. Moreover, for some organs, such as the mandible and the submandibular glands, DSC values were significantly higher in EID-CT than with PCD-CT. These findings suggest that while PCD-CT, especially with combined energies, can enhance inter-expert agreement for specific OARs, conventional EID-CT may still provide higher agreement in some cases. Nevertheless, it is unclear if the small improvements in agreement observed for PCD-CT are clinically relevant in the radiotherapy workflow.

In the future, a PCD-CT-only workflow could enable a) acquisition of a single contrast-enhanced examination without the need for additional diagnostic imaging, b) virtual contrast subtraction to generate images more suitable for planning, c) lower radiation exposure, d) improved material characterization with more accurate dose calculation, and e) reduced artifacts. Future studies could further explore potential advantages in target segmentation by leveraging more reconstructions. Many of these features are also achievable with dual-energy CT (DECT), however at a cost of higher imaging dose; moreover, recent investigations are showing better performance of PCD-CT over DECT for certain applications, although further validation is required [32].

This study has several limitations, including the relatively small number of patients and physicians involved in the contouring process, as well as the fact that the two CT scans were performed at different appointments, which could have influenced patient positioning and anatomy. Furthermore, this study did not evaluate the ultra-high-resolution mode of PCD-CT, which provides a higher spatial resolution than the scan modes tested here [33]. Additionally, only two of many possible PCD-CT energies and reconstructions were investigated. Despite these limitations, to our knowledge this is the first prospective study to demonstrate PCD-CT-based OAR and CTV delineation results, possibly paving the way for future clinical use of PCD-CT in radiation therapy.

In conclusion this study demonstrates, for the first time in clinically obtained, real-world images, that PCD-CT provides delineation agreement at least comparable to conventional EID-CT in patients with head-and-neck cancer, even in challenging scenarios involving in-field metal artifacts. The potential advantages of PCD-CT, including reduced radiation dose and enhanced diagnostic image quality, underscore its promise in clinical applications.

## CRediT authorship contribution statement

**Niccolò Bertini:** Conceptualization, Project administration, Data curation, Software, Formal analysis, Investigation, Methodology, Writing – original draft, Writing – review & editing. **Hubert S. Gabryś:** Conceptualization, Project administration, Data curation, Software, Formal analysis, Investigation, Methodology, Writing – original draft, Writing – review & editing. **Hatem Alkadhi:** Software, Resources, Methodology, Writing – review & editing. **Lotte Wilke:** Supervision, Data curation, Software, Methodology, Writing – review & editing. **Patrick Wohlfahrt:** Methodology, Writing – review & editing. **Serena Psoroulas:** Data curation, Writing – review & editing. **Eugenia Vlaskou:** Supervision, Data curation, Software, Methodology, Writing – review & editing. **Laura Motisi:** Supervision, Data curation, Software, Methodology, Writing – review & editing. **Matthias Guckenberger:** . **Stephanie Tanadini-Lang:** Supervision, Data curation, Software, Methodology, Writing – review & editing. **Panagiotis Balermpas:** Conceptualization, Project administration, Data curation, Software, Methodology, Writing – original draft, Writing – review & editing.

## Funding

None.

## Declaration of competing interest

The authors declare the following financial interests/personal relationships which may be considered as potential competing interests: The department of radiation oncology, Zurich University Hospital, has a research agreement on photon counting CT with Siemens Healthineers. Patrick Wohlfahrt is an employee within the research and development team of Siemens Healthineers. The Department of Diagnostic and Interventional Radiology has research agreements with Bayer, Canon, Guerbet and Siemens. H.A. received speaker fee’s from Siemens. Niccolo’ Bertini, declares no conflicts of interest.
