# Supplementary material for: First implementation of photon-counting dector computed tomography for optimizing segmentation in head-and-neck cancer radiotherapy
Source: Phys Imaging Radiat Oncol. 2025 Nov 13;36:100864. doi: 10.1016/j.phro.2025.100864 (PMC12702377; doi:10.1016/j.phro.2025.100864)

**Supplement**

**Supplementary Table S1**. DSC comparison between experts using a native energy-integrating single-energy CT (EID-CT), a virtual monoenergetic image of 50 keV derived from photon-counting CT (PCD-CT 50 keV), a virtual monoenergetic image of 140 keV derived from photon-counting CT (PCD-CT 140 keV), and *p*-values adjusted with the Holm method.

| **ROI** | **Modality A** | **Modality B** | **DSC A** | **DSC B** | **p-value** |
| --- | --- | --- | --- | --- | --- |
| CTV | EID-CT | PCD-CT  50 keV | 0.579 | 0.563 | 1.000 |
| CTV | EID-CT | PCD-CT  140 keV | 0.579 | 0.559 | 1.000 |
| CTV | PCD-CT  50 keV | PCD-CT  140 keV | 0.563 | 0.559 | 1.000 |
| Buccal Mucosa | EID-CT | PCD-CT  50 keV | 0.515 | 0.548 | 0.369 |
| Buccal Mucosa | EID-CT | PCD-CT  140 keV | 0.515 | 0.505 | 0.686 |
| Buccal Mucosa | PCD-CT  50 keV | PCD-CT  140 keV | 0.548 | 0.505 | 0.250 |
| Lips | EID-CT | PCD-CT  50 keV | 0.559 | 0.613 | **0.001** |
| Lips | EID-CT | PCD-CT  140 keV | 0.559 | 0.574 | 0.326 |
| Lips | PCD-CT  50 keV | PCD-CT  140 keV | 0.613 | 0.574 | **0.012** |
| Mandible | EID-CT | PCD-CT  50 keV | 0.856 | 0.841 | 0.098 |
| Mandible | EID-CT | PCD-CT  140 keV | 0.856 | 0.834 | **0.010** |
| Mandible | PCD-CT  50 keV | PCD-CT  140 keV | 0.841 | 0.834 | 0.335 |
| Oral Cavity | EID-CT | PCD-CT  50 keV | 0.824 | 0.855 | **0.003** |
| Oral Cavity | EID-CT | PCD-CT  140 keV | 0.824 | 0.849 | **0.016** |
| Oral Cavity | PCD-CT  50 keV | PCD-CT  140 keV | 0.855 | 0.849 | 0.520 |
| Parotid Glands | EID-CT | PCD-CT  50 keV | 0.815 | 0.831 | 0.171 |
| Parotid Glands | EID-CT | PCD-CT  140 keV | 0.815 | 0.834 | 0.126 |
| Parotid Glands | PCD-CT  50 keV | PCD-CT  140 keV | 0.831 | 0.834 | 0.749 |
| Pharyngeal  Constrictors | EID-CT | PCD-CT  50 keV | 0.513 | 0.544 | 0.289 |
| Pharyngeal  Constrictors | EID-CT | PCD-CT  140 keV | 0.513 | 0.473 | 0.289 |
| Pharyngeal  Constrictors | PCD-CT  50 keV | PCD-CT  140 keV | 0.544 | 0.473 | **0.030** |
| Submandibular  Glands | EID-CT | PCD-CT  50 keV | 0.841 | 0.823 | 0.120 |
| Submandibular  Glands | EID-CT | PCD-CT  140 keV | 0.841 | 0.799 | **0.001** |
| Submandibular  Glands | PCD-CT  50 keV | PCD-CT  140 keV | 0.823 | 0.799 | 0.075 |

**Supplementary Table S2**. Hausdorff Distance in mm (HD) comparison between experts using a native energy-integrating single-energy CT (EID-CT), a virtual monoenergetic image of 50 keV derived from photon-counting CT (PCD-CT 50 keV), a virtual monoenergetic image of 140 keV derived from photon-counting CT (PCD-CT 140 keV), and *p*-values adjusted with the Holm method.

| **ROI** | **Modality A** | **Modality B** | **HD A** | **HD B** | **p-value** |
| --- | --- | --- | --- | --- | --- |
| CTV | EID-CT | PCD-CT  50 keV | 25.9 | 24.7 | 1.000 |
| CTV | EID-CT | PCD-CT  140 keV | 25.9 | 24.0 | 1.000 |
| CTV | PCD-CT  50 keV | PCD-CT  140 keV | 24.7 | 24.0 | 1.000 |
| Buccal Mucosa | EID-CT | PCD-CT  50 keV | 14.6 | 14.7 | 1.000 |
| Buccal Mucosa | EID-CT | PCD-CT  140 keV | 14.6 | 14.4 | 1.000 |
| Buccal Mucosa | PCD-CT  50 keV | PCD-CT  140 keV | 14.7 | 14.4 | 1.000 |
| Lips | EID-CT | PCD-CT  50 keV | 13.7 | 12.3 | 0.100 |
| Lips | EID-CT | PCD-CT  140 keV | 13.7 | 12.4 | 0.104 |
| Lips | PCD-CT  50 keV | PCD-CT  140 keV | 12.3 | 12.4 | 0.845 |
| Mandible | EID-CT | PCD-CT  50 keV | 9.17 | 11.75 | **0.026** |
| Mandible | EID-CT | PCD-CT  140 keV | 9.17 | 11.56 | **0.028** |
| Mandible | PCD-CT  50 keV | PCD-CT  140 keV | 11.75 | 11.56 | 0.861 |
| Oral Cavity | EID-CT | PCD-CT  50 keV | 16.4 | 12.1 | **<.001** |
| Oral Cavity | EID-CT | PCD-CT  140 keV | 16.4 | 12.4 | **<.001** |
| Oral Cavity | PCD-CT  50 keV | PCD-CT  140 keV | 12.1 | 12.4 | 0.663 |
| Parotid Glands | EID-CT | PCD-CT  50 keV | 12.4 | 11.0 | 0.178 |
| Parotid Glands | EID-CT | PCD-CT  140 keV | 12.4 | 11.2 | 0.240 |
| Parotid Glands | PCD-CT  50 keV | PCD-CT  140 keV | 11.0 | 11.2 | 0.738 |
| Pharyngeal  Constrictors | EID-CT | PCD-CT  50 keV | 14.7 | 13.5 | 0.632 |
| Pharyngeal  Constrictors | EID-CT | PCD-CT  140 keV | 14.7 | 16.4 | 0.632 |
| Pharyngeal  Constrictors | PCD-CT  50 keV | PCD-CT  140 keV | 13.5 | 16.4 | 0.227 |
| Submandibular  Glands | EID-CT | PCD-CT  50 keV | 6.33 | 7.52 | 0.1044 |
| Submandibular  Glands | EID-CT | PCD-CT  140 keV | 6.33 | 7.79 | 0.0588 |
| Submandibular  Glands | PCD-CT  50 keV | PCD-CT  140 keV | 7.52 | 7.79 | 0.6849 |

**Supplementary Table S3**. Mean Distance to Agreement in mm (MDA) comparison between experts using a native energy-integrating single-energy CT (EID-CT), a virtual monoenergetic image of 50 keV derived from photon-counting CT (PCD-CT 50 keV), a virtual monoenergetic image of 140 keV derived from photon-counting CT (PCD-CT 140 keV), and *p*-values adjusted with the Holm method.

| **ROI** | **Modality A** | **Modality B** | **MDA A** | **MDA B** | **p-value** |
| --- | --- | --- | --- | --- | --- |
| CTV | EID-CT | PCD-CT  50 keV | 6.06 | 6.12 | 1.000 |
| CTV | EID-CT | PCD-CT  140 keV | 6.06 | 5.99 | 1.000 |
| CTV | PCD-CT  50 keV | PCD-CT  140 keV | 6.12 | 5.99 | 1.000 |
| Buccal Mucosa | EID-CT | PCD-CT  50 keV | 2.37 | 2.48 | 0.8275 |
| Buccal Mucosa | EID-CT | PCD-CT  140 keV | 2.37 | 2.51 | 0.8275 |
| Buccal Mucosa | PCD-CT  50 keV | PCD-CT  140 keV | 2.48 | 2.51 | 0.8275 |
| Lips | EID-CT | PCD-CT  50 keV | 2.33 | 2.06 | **0.0469** |
| Lips | EID-CT | PCD-CT  140 keV | 2.33 | 2.31 | 0.8430 |
| Lips | PCD-CT  50 keV | PCD-CT  140 keV | 2.06 | 2.31 | **0.0469** |
| Mandible | EID-CT | PCD-CT  50 keV | 0.717 | 0.848 | **0.048** |
| Mandible | EID-CT | PCD-CT  140 keV | 0.717 | 0.905 | **0.006** |
| Mandible | PCD-CT  50 keV | PCD-CT  140 keV | 0.848 | 0.905 | 0.378 |
| Oral Cavity | EID-CT | PCD-CT  50 keV | 2.56 | 2.04 | **0.0046** |
| Oral Cavity | EID-CT | PCD-CT  140 keV | 2.56 | 2.14 | **0.0218** |
| Oral Cavity | PCD-CT  50 keV | PCD-CT  140 keV | 2.04 | 2.14 | 0.5046 |
| Parotid Glands | EID-CT | PCD-CT  50 keV | 1.35 | 1.26 | 0.6274 |
| Parotid Glands | EID-CT | PCD-CT  140 keV | 1.35 | 1.23 | 0.5643 |
| Parotid Glands | PCD-CT  50 keV | PCD-CT  140 keV | 1.26 | 1.23 | 0.7563 |
| Pharyngeal  Constrictors | EID-CT | PCD-CT  50 keV | 1.84 | 1.56 | 0.4954 |
| Pharyngeal  Constrictors | EID-CT | PCD-CT  140 keV | 1.84 | 2.16 | 0.4954 |
| Pharyngeal  Constrictors | PCD-CT  50 keV | PCD-CT  140 keV | 1.56 | 2.16 | 0.0776 |
| Submandibular  Glands | EID-CT | PCD-CT  50 keV | 0.91 | 1.02 | 0.2146 |
| Submandibular  Glands | EID-CT | PCD-CT  140 keV | 0.91 | 1.11 | **0.0198** |
| Submandibular  Glands | PCD-CT  50 keV | PCD-CT  140 keV | 1.02 | 1.11 | 0.2536 |

**Supplementary Table S4**. Detailing results of confidence contouring rating

|  | EID-CT | | PCD-CT | |
| --- | --- | --- | --- | --- |
|  | Mean | Range (min-max) | Mean | Range |
| CTV |  |  |  |  |
| Observer 1 | 7,2 | 6 – 9 | 8,6 | 7 – 10 |
| Observer 2 | 7,3 | 5 – 9 | 8,1 | 6 – 10 |
| Observer 3 | 7,7 | 6 – 9 | 8,1 | 7 – 10 |
| Total | 7,4 | 5 – 9 | 8,3 | 6 – 10 |
| Mandible |  |  |  |  |
| Observer 1 | 10 | 10 – 10 | 10 | 10 – 10 |
| Observer 2 | 9,9 | 9 – 10 | 10 | 10 – 10 |
| Observer 3 | 7,9 | 6 – 9 | 9,1 | 8 – 10 |
| Total | 9,3 | 6 – 10 | 9,7 | 8 – 10 |
| Lips |  |  |  |  |
| Observer 1 | 7 | 6 – 9 | 8,5 | 7 – 10 |
| Observer 2 | 6,3 | 4 – 8 | 7,4 | 5 – 10 |
| Observer 3 | 7,3 | 6 – 8 | 8,5 | 8 – 10 |
| Total | 6,9 | 4 – 9 | 8,1 | 5 – 10 |
| Other OARs |  |  |  |  |
| Observer 1 | 8,2 | 7 – 9 | 9 | 8 – 10 |
| Observer 2 | 7,6 | 6 – 9 | 8,4 | 6 – 10 |
| Observer 3 | 8,5 | 7 – 10 | 9,1 | 8 – 10 |
| Total | 8,1 | 6 – 10 | 8,8 | 6 – 10 |

This table presents the results of confidence contouring ratings across three anatomical structures (CTV, Mandible, and Lips) by three observers using two imaging techniques: EID-CT and PCD-CT. The data includes mean ratings and their respective ranges (minimum-maximum).

Supplementary Figure S5a: Computed Tomography Dose Index (CTDIvol)

This figure illustrates the CTDIvol (in mGy) for each patient across the two imaging methods: EID-CT and PCD-CT. The blue bars represent EID-CT values, while the orange bars represent PCD-CT values.
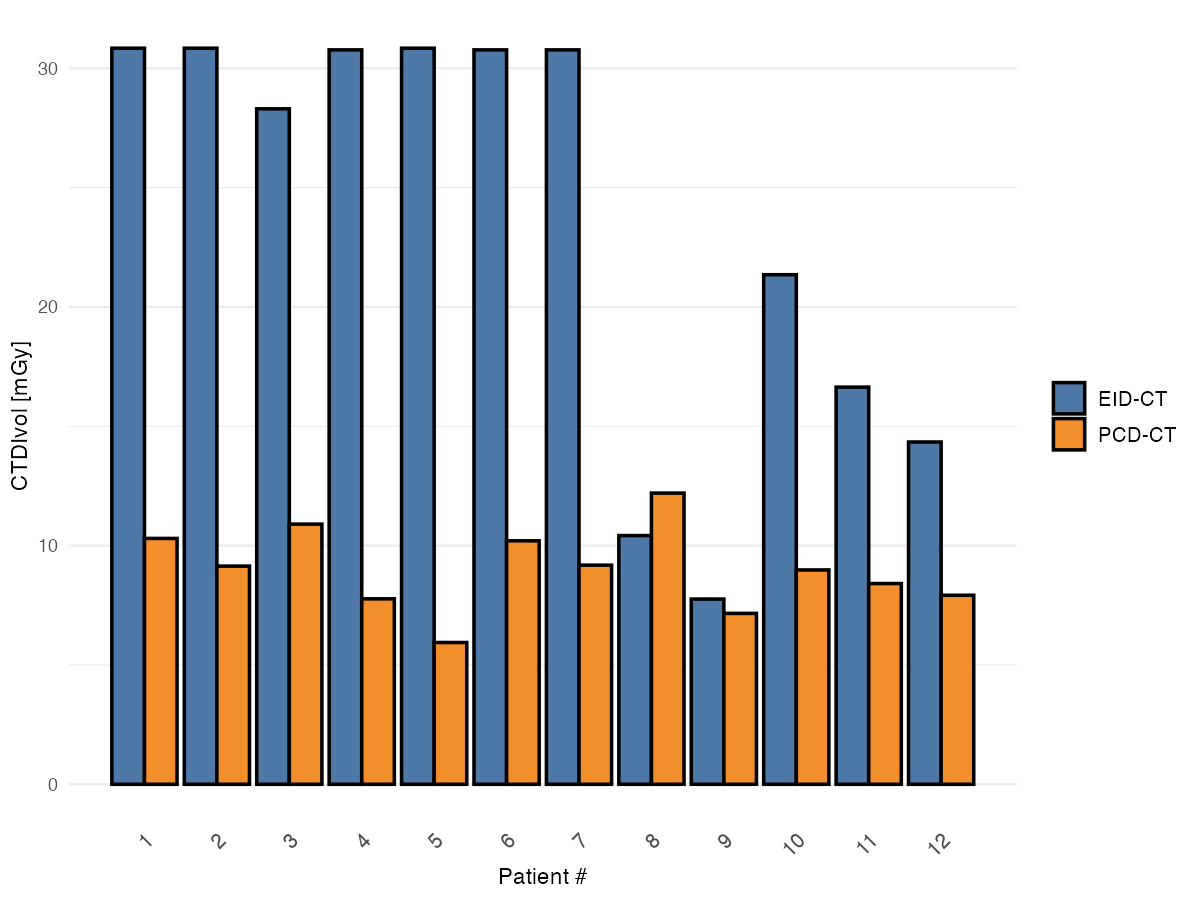


Supplementary Figure S5b: Dose Length Product (DLP)

This figure presents the Dose Length Product (DLP, in mGy·cm) for all patients for both EID-CT and PCD-CT technique.


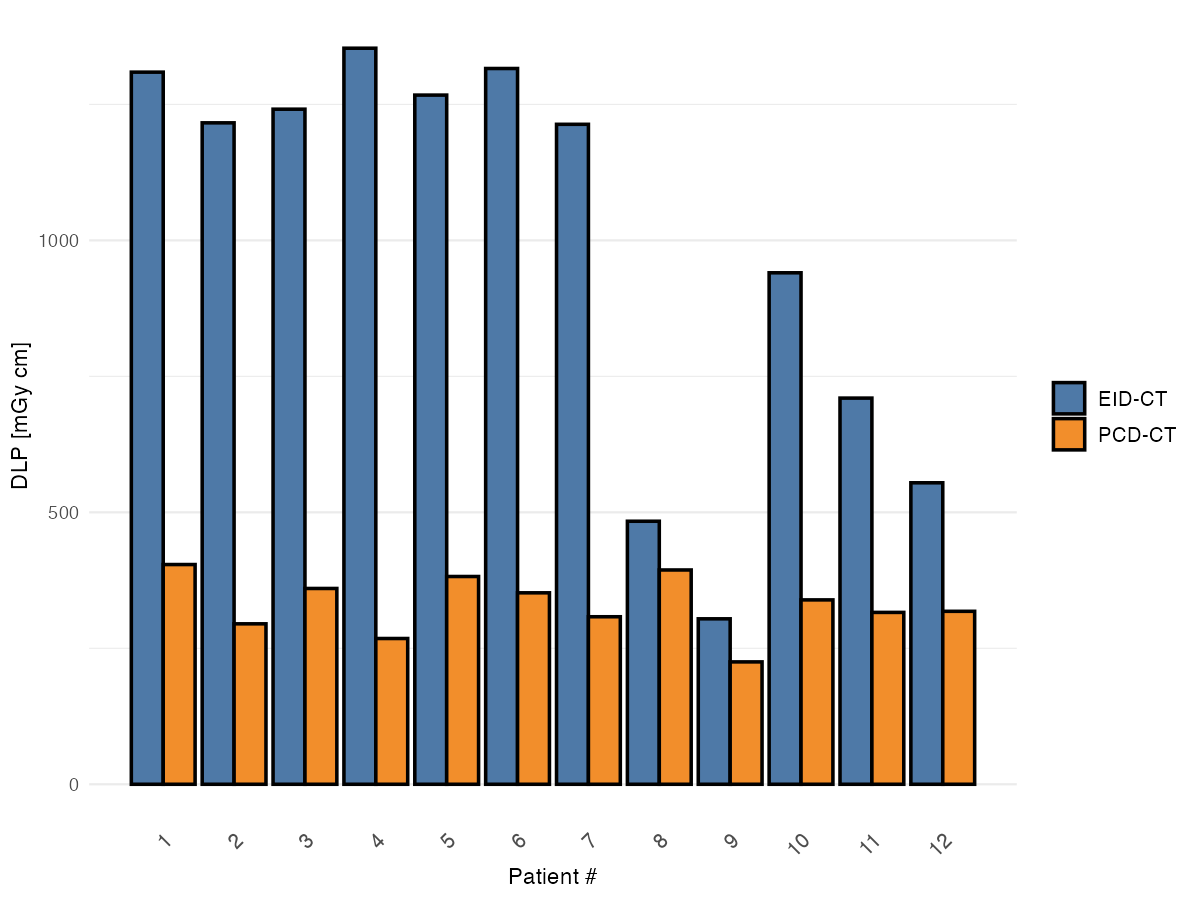


Supplementary Figure S5c: Total mAs

The total mAs (tube current-time product) for each patient is shown in this figure for both EID-CT and PCD-CT methods.
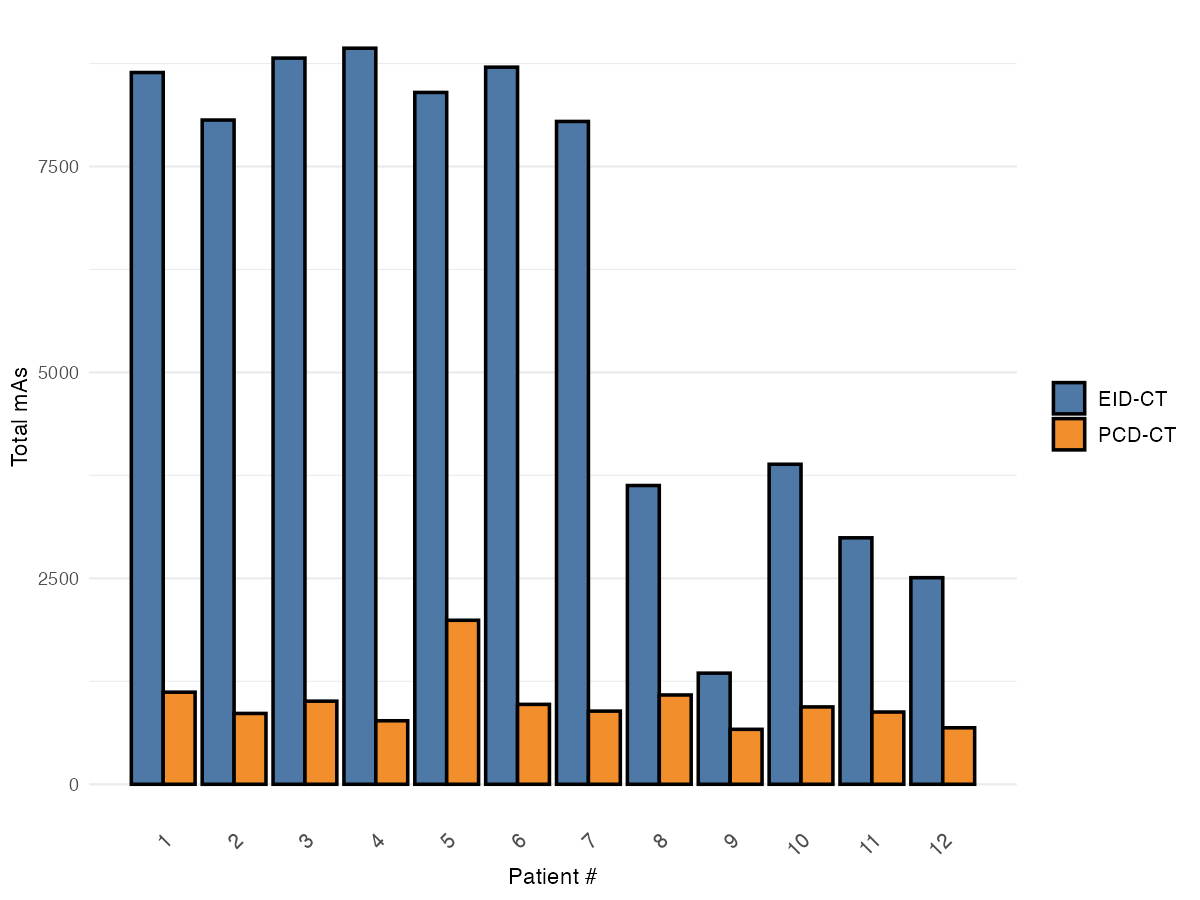


Supplementary Figure S6 Examples of CT slices containing target volume and metal for each patient.
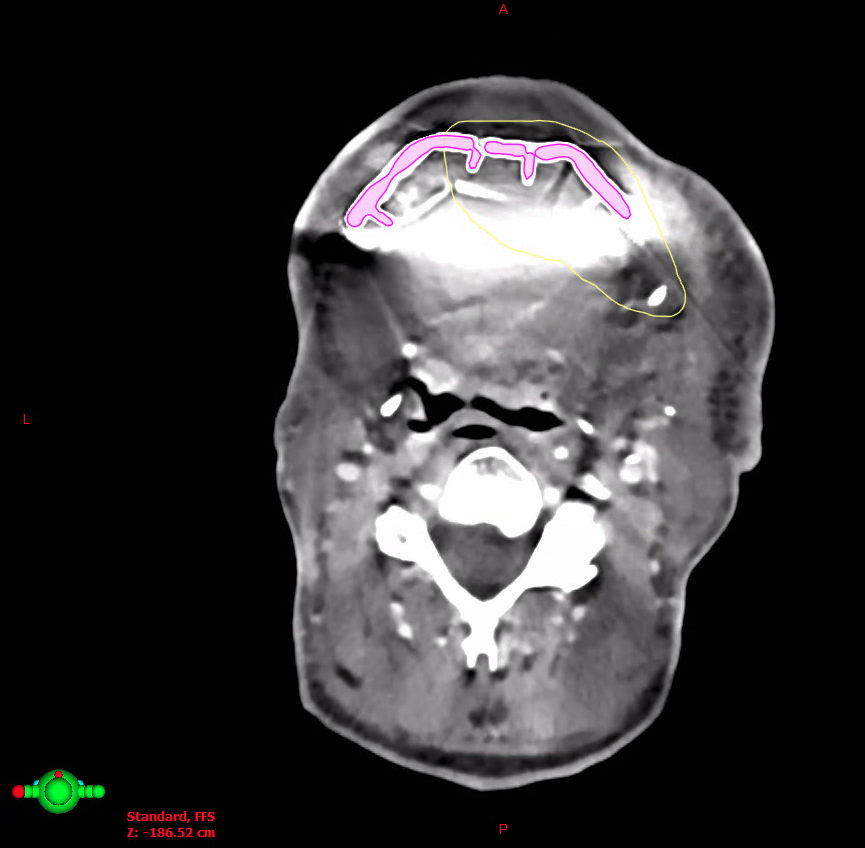


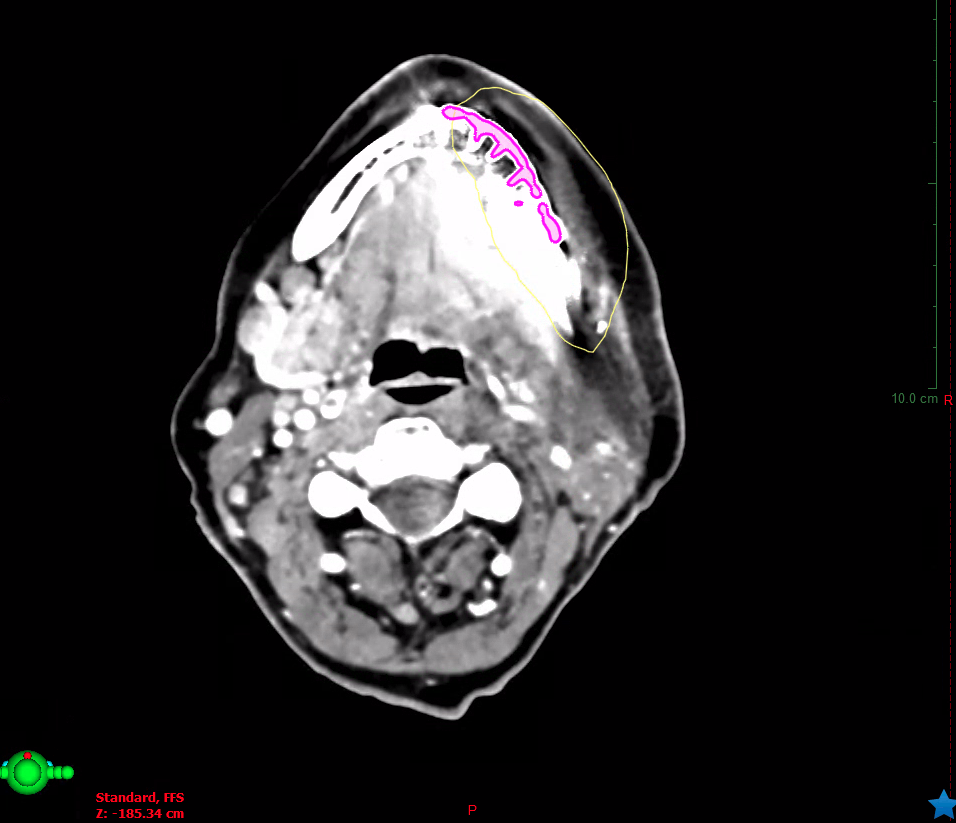

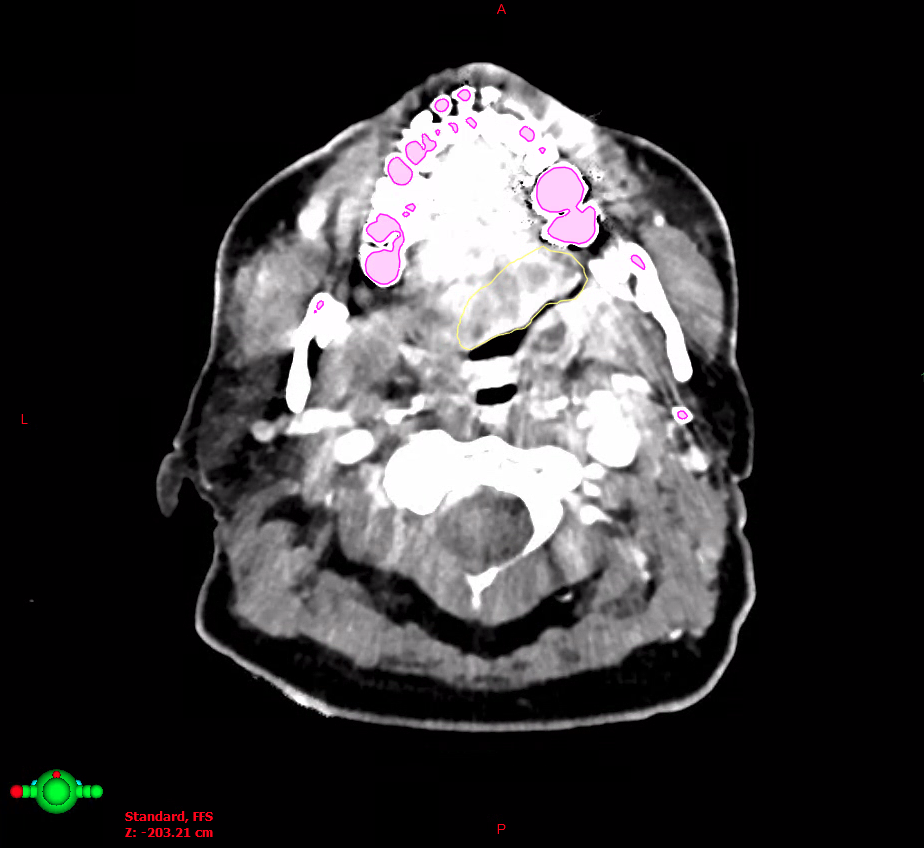

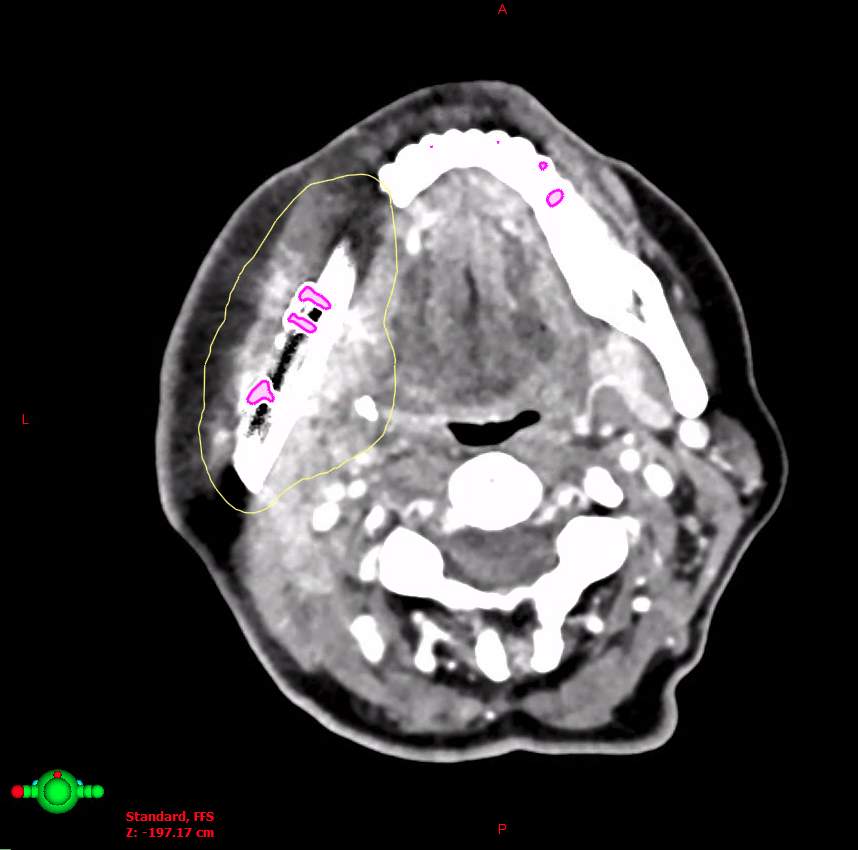

Supplement: Supplementary Data 1 [file mmc1.docx]
